# Gas; Ether; Chloroform

**Published:** 1901-04-06

**Authors:** 


### GAS; ETHER; CHLOROFORM.

In a lecture recently delivered Dr. Frederic W. Hewitt[1] advocates the use of the three anæsthetics, gas, ether, and chloroform, given in the above sequence as a most satisfactory means of producing and maintaining anæsthesia.

He says that, of all the known systems of inducing anæsthesia, there can be no doubt that that of administering nitrous oxide and ether in succession, introduced many years ago by Mr. Clover, is the best. But there are certain disadvantageous after-effects connected with the administration, and especially the prolonged administration, of ether, viz. the taste and smell, experienced by the patient, the nausea and vomiting, and the bronchial and pulmonary sequelæ. By the use of gas and ether, however, to put the patient "under," and by then continuing the anæsthesia with chloroform, many advantages are gained. Loosely speaking, we may say that "gas" is given for the patient, ether for the anæsthetist, and chloroform for the surgeon. Nitrous oxide is advantageous to the patient because by its means he is saved the disagreeable sensations attending the respiration of ether vapour; ether is advantageous to the anæsthetist because of its great safety, and its stimulant action upon the respiration and circulation; and chloroform is advantageous to the surgeon because of the comparative quietude of the patient and the comparative freedom from venous engorgement and hæmorrhage during the administration. Moreover the principal risk of chloroform is that which is incurred during the second stage of anæsthetisation—a stage which in certain subjects is characterised by excitement, rigidity, and struggling. Now by the sequence recommended this risk is wholly eliminated, for the struggling stage is passed over while the patient is under nitrous oxide and ether, with which anæsthetics there is, practically speaking, no such risk. We are therefore able to obtain the advantages of chloroform without experiencing one of its chief disadvantages. In the same way we avoid the chief disadvantages of ether, for the quantity used is insufficient to prejudicially affect the recovery period. At any ate Dr. Hewitt says: "Of this I am convinced, that there is no plan at present known of inducing and maintaining surgical anæsthesia which has so many advantages and so few disadvantages as the one I now describe." There are, however, certain precautions to be observed. One must never change from ether to chloroform while the patient is struggling. Then one must be careful never to effect the change unless there is some evidence, at the moment of change, that the patient is not profoundly anæsthetised by the ether. Thus it is a good plan to administer ether till the corneal reflex has just disappeared, to remove the ether inhaler for a few breaths of air, to allow a slight cough to occur in order to free the larynx of any mucus that may have entered it, to watch for the first indication of returning corneal reflex, and then to begin with the chloroform inhalation. This cough is important, for if chloroform should be started whilst the larynx contains mucus considerable respiratory difficulty may arise. Again, it is most important in abdominal cases to effect the change before the operation is begun, for as this change should only be made during a comparatively light anæsthesia, and as this condition may be associated with rigidity or movement, much inconvenience to the surgeon may be caused by changing when the operation is in progress. When once the patient has been placed well under ether, and has been allowed to clear the larynx, it is not necessary to maintain a very profound chloroform narcosis.

[1] Lancet, March 30.

